# Lost in transcription – how accurately are we documenting the surgical ward round?

**DOI:** 10.1111/ans.70109

**Published:** 2025-04-09

**Authors:** Ellie C. Treloar, Ying Y. Ting, Martin H. Bruening, Jessica L. Reid, Suzanne Edwards, Emma L. Bradshaw, Jesse D. Ey, Matthias Wichmann, Matheesha Herath, Guy J. Maddern

**Affiliations:** ^1^ Department of Surgery The University of Adelaide, The Queen Elizabeth Hospital Woodville South Australia Australia; ^2^ Department of General Surgery Mount Gambier and Districts Health Service Mount Gambier South Australia Australia

**Keywords:** accuracy, documentation, surgery, surgical case notes, ward rounds

## Abstract

**Background:**

Ward rounds are crucial to providing high‐quality patient care in hospitals.

Ward round quality is strongly linked to patient outcomes, yet ward round best practice is severely underrepresented in the literature. Accurate and thorough ward round documentation is essential to improving communication and patient outcomes.

**Methods:**

A prospective observational cohort study was performed by reviewing 135 audio‐visual recordings of surgical ward rounds over 2 years at two hospitals. Recordings were transcribed, and an external reviewer stratified discussion points as Major, Minor, or Not Significant. Discussion was compared to the ward round note to assess the accuracy of documentation based on bedside discussion. The primary endpoint was the accuracy of Major discussion in the patient case notes. Secondary objectives involved investigating variables that may have impacted accuracy (e.g., patient age, sex, length of stay in hospital, and individual clinicians).

**Results:**

Nearly one third (32.4%) of important (Major) spoken information regarding plans and patient care in the ward round was omitted from the patients' written medical record. Further, 11% of patient case notes contained significant errors. Patient age (*P* = 0.04), the day of the week on which the ward round occurred (*P* = 0.05) and who the scribing intern was (*P* ≤ 0.001) were found to impact documentation accuracy. There was a large variation in interns documenting ability (35.5%–88.9% accuracy).

**Conclusions:**

This study highlighted that a significant portion of important discussion conducted during the ward round is not documented in the case note. These results suggest that system‐wide change is needed to improve patient safety and outcomes.

## Graphical abstract

Accurate and precise patient case notes.

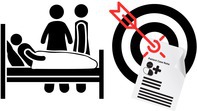

## Introduction

Ward rounds are integral to providing high‐quality patient care.^1^, ^2^ Conducted daily, they provide opportunities for teams to systematically review each patient in their care.^3^, ^4^ During rounds, the team addresses patient concerns, evaluates progress, and reviews recent events in order to establish a care plan regarding diagnosis, treatment, or discharge.^3^, ^5^ While the surgical ward round is typically led by a senior member of the team,^6^ the events occurring during the round are often documented by the most junior team member, usually the intern or medical student.^1^, ^4^, ^7^, ^8^, ^9^ These case notes act as a communication tool between teams caring for the patient and are a permanent medicolegal document.^10^, ^11^

The quality of the ward round relies on the quality of information exchanged between members of the team.^12^ This includes ensuring that decisions made and discussions had on the ward round are accurately and legibly recorded in the patient's case notes.^13^, ^14^ Current best practice recommends that case note documentation should be methodical and include: a timestamp, all relevant clinical findings reported in a structured manner, a record of decisions made, any drug prescribed, investigations performed, and the identity of who made the record.^13^, ^15^ Comprehensive and accurate notes are important for scientific research, indexing for funding, and overall adherence to good clinical practice.^12^, ^16^ Inaccurate or missing documentation is associated with increased adverse events and prolonged hospital stay.^7^, ^12^ In time pressured and complex environments such as the surgical ward round, it can be challenging to maintain good documentation.^10^, ^17^, ^18^ Chaotic environments increase the likelihood of miscommunication and errors, especially when the role of documenting is routinely left to the most junior member of the team.^1^, ^9^, ^17^

Patient care is dependent on the accuracy, legibility, and quality of patient case notes in the ward round. However, ward round ‘best practice’ and methods to improve documentation is severely underrepresented in the literature.^12^, ^13^ The primary objective of this study was therefore to determine the accuracy of surgical ward round case note documentation through audio‐visual analysis. The secondary objective was to determine which factors affected this accuracy (e.g., patient age, sex, length of stay, day of the week, clinician experience, and individual senior clinician leadership).

## Materials and methods

This study was conducted in the General Surgical Units of two hospitals over 24 months and was approved by the Central Adelaide Local Health Network Human Research Ethics Committee (#12993). The aim of the study was to assess 135 audio‐visual recordings of surgical ward rounds and compare the discussion occurring in the ward round to the patient case notes. The purpose of this comparison was to identify how accurately the patient case notes were documented, determine the presence of omitted and incorrect documentation, and understand the factors that may have affected this.

To capture the recordings for assessment, surgical ward round encounters between consenting clinicians and patients were audio‐visually recorded using a small camera (GoPro) placed in an unhidden but non‐obtrusive position. Eligible patients included adults admitted to the surgical wards of the two participating hospitals who had a good comprehension of the English language and the capacity to consent; these patients were selected utilizing convenience sampling. All surgical team members were eligible for inclusion. All consenting patients and staff members were provided with information regarding the potential risks and risk mitigation strategies regarding privacy concerns with GoPro use. To adhere to ethical standards, only the study investigators viewed ward round videos. These were then transcribed verbatim in a de‐identified manner in order to compare discussion to patient case notes.

The transcriptions of the ward rounds were then reviewed and grouped into ‘discussion points’ (e.g., questions and answers). Grouped transcripts were sent to an external reviewer (senior clinician with over 20 years of experience) to assign each discussion point a level of importance. The designations were then reviewed by multiple blinded clinicians to ensure consensus agreement and conflicts were resolved by discussion. The included ‘Major’, ‘Minor’, or ‘Not Significant’, in the context of patient care (e.g., The importance of a discussion point being documented in the patients' case notes). Prior to study commencement, definitions for ‘Major’, ‘Minor’, and ‘Not Significant’ were developed through a Modified Delphi process with a panel consisting of surgeons, junior doctors, clinical academics, scientists, and lay persons. The definitions for each of these are as follows:

1. ‘Major’: defined as discussion components that would likely have a direct or serious impact on patient progress or delay their discharge if not documented (e.g., drain output, diet change, and booking for theatre).
2. ‘Minor’: defined as a point of discussion that would cause minimal or no harm if it was not documented, but was still relevant to patient care (e.g., verbalisation of decision‐making processes rather than the final decision made).
3. ‘Not significant’: any discussion that was irrelevant to the patient's progression or care.

The transcriptions were then compared to patient case notes by two study investigators. Points of discussion were classified as either:

1. ‘Recorded Correctly’: the discussion was documented accurately
2. ‘Recorded Incorrectly’: the discussion was documented in contradictory fashion to the discussion
3. ‘Not Recorded’: the discussion was omitted from the documentation

Following classification, the percentage of discussion points accurately recorded in patient notes were calculated. The proportion, mean, and standard deviation of ‘Recorded correctly’, ‘Recorded incorrectly’ and ‘Not recorded’ for each ‘Major’, ‘Minor’, and ‘Not Significant’ point was then determined. To address the primary objective (determining the accuracy of patient case notes), the number of ‘Major’, ‘Minor’ and ‘Not significant’ points in each audio‐visual recording were tallied. To address the secondary objective (determining the factors affecting documentation accuracy), two multivariable linear mixed‐effect models were performed. These were performed between outcomes determined on an *a priori* basis by the literature: percentage of ‘Major’ and ‘Minor’ and the covariates: patient age, location (rural or metropolitan hospital), patient sex (male, female), day of the week of the encounter, length of stay at the time of the ward round, who the clinician was, who the intern was, and intern experience.

## Results

### Demographics

A total of 135 ward round encounters were recorded, involving 78 patients. There were six patients who declined participation, with no patients withdrawing once consented. No staff declined participation or withdrew. Of the 78 patients participating in the study, there were 40/78 (51%) males and 38/78 (49%) females, with the mean age of 62 (SD: 15.2). The median length of stay in the hospital at the time of video recording the ward round was 3 days (IQR: 2, 10). Each patient had between one and 10 encounters recorded. There were 11 senior clinicians and 17 interns who participated. The clinicians led a median of seven ward rounds (IQR: 4, 13) and each intern documented a median of seven ward rounds (IQR: 3, 9). Given the rotational basis of junior doctors, the interns were on their rotation in the surgical unit for a median of 32 days (IQR: 25.5, 44) and on their internship year for a median of 170 days (IQR: 33, 235) on the day the ward round encounter was recorded. There was an average of 16 discussion points in each ward round (SD: 7.2), with most points discussed deemed ‘Major’.

### Accuracy of patient case note documentation

Of the 135 ward round encounters, there were 1230 discussion items marked as ‘Major’, 545 as ‘Minor’, and 381 discussion points as ‘Not significant’. Of the discussion items considered ‘Major’, documentation was accurate in the patient case notes 67.6% (832/1230) of the time, incorrect 4.1% (50/1230) of the time, and entirely omitted 28.3% (348/1230) of the time. Of the discussion items considered ‘Minor’, they were documented accurately 55% (300/545) of the time, incorrectly 4.4% of the time (24/545), and omitted 40.6% of the time (569/545). This is depicted in Table 1. Examples of the incorrect documentation can be found in Table 2.

**Table 1:** Accuracy of patient case note documentation

	Recorded	Recorded incorrectly	Not recorded
Major	832/1230 (67.6%)	50/1230 (4.1%)	348/1230 (28.3%)
Minor	300/545 (55.0%)	24/545 (4.4%)	221/545 (40.6%)
Total	1132/ 1775 (63.8%)	74 / 1775 (4.2%)	569/1775 (32.0%)

**Table 2:** Discrepancies between discussion in the ward round captured by audio‐visual footage and the documented patient case note

Transcript of discussion in the ward round	Extraction of patient case note for the same ward round
‘NGT 100 mL yesterday…20 mL through abdominal drain’	NG output 600 mL yesterday, Abdo drain 280 mL yesterday
‘The urinary drain, I think we should take that one out because every line is a risk for potential future infection’	‘Catheter and drain to stay insitu’
‘Refer for ERCP on Monday’	‘Referral to gastroenterology for ERCP on Wednesday’
‘Everything went really well yesterday, so you had the ERCP procedure’	“For ERCP and likely stenting today”
‘I still feel nauseous… it's very uncomfortable’	‘Denies nausea or vomiting’ and ‘Feeling well in herself’

### Factors affecting documentation

**Fig. 1:**
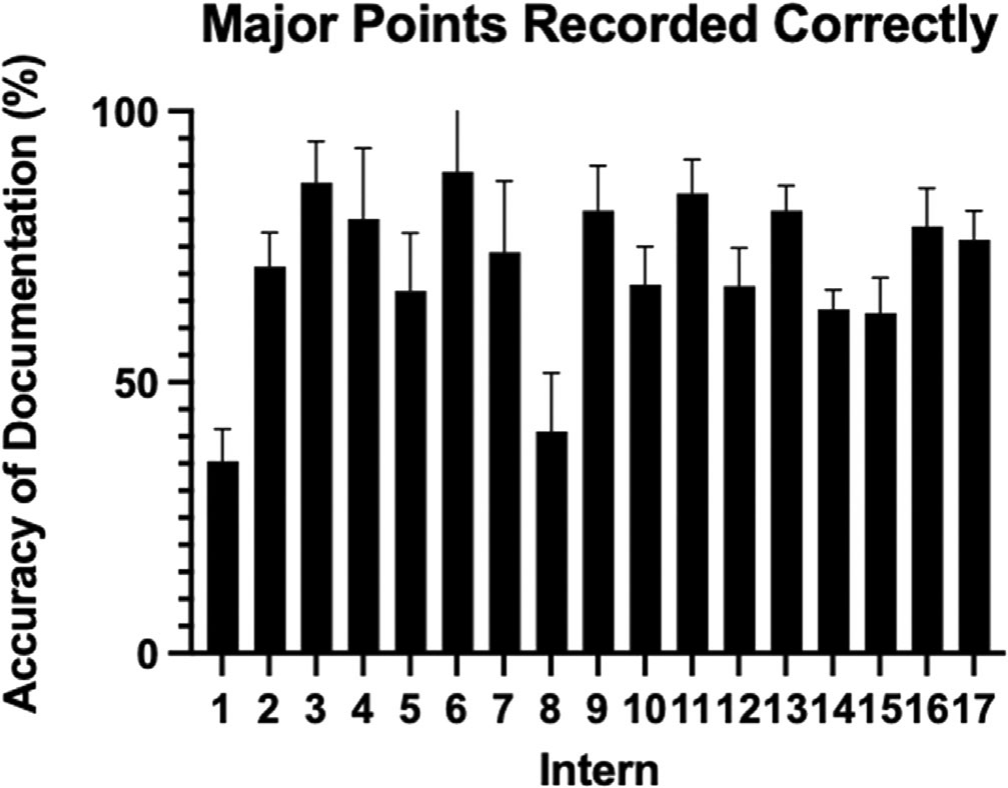
Bar graph displaying documentation rates of ‘Major’ points of 17 included interns.

There was a significant association between ‘Major’ points recorded correctly and patient age (*P* = 0.04), as well as day of the week (*P* = 0.05), and who was scribing (*P* ≤ 0.001). Patient sex, how long the patient had been in hospital prior to the ward round recording, location (rural or metropolitan hospital), seniority of the clinician leading the ward round, start date of internship, and start date of rotation did not impact the accuracy of documentation of ‘Major’ points. There was significant association between ‘Minor’ points recorded correctly and location (*P* = 0.03), and who was scribing (*P* = 0.005); no other covariates had an association with ‘Minor’ points. Of the 15 scribing interns, documentation accuracy ranged from 35.5% to 88.9%. The differences between the documentation accuracy of ‘Major’ points are displayed in Figure 1.

## Discussion

This study assessed the accuracy of patient case note documentation in surgical ward rounds.

The primary findings indicated that nearly a third of ‘Major’ discussion (i.e., discussion that would have a direct or serious impact on patient care if not documented) was omitted from the patients' case note. These omitted components of documentation involved critical information about patient care including diagnosis, necessary investigations, medical management, patient concerns, and discharge planning. In addition to the omitted documentation, 4.2% of the patient's overall case notes contained incorrect documentation; and 11% of patients enrolled in the study had at least one incorrectly documented plan. Inaccurate documentation can impact decision making resulting in incorrect investigations or treatments being ordered. This can subsequently result in prolonged or missed diagnosis, increased risk of complication and discomfort for the patient.^19^, ^20^

There are several possible reasons to explain the presence of inaccurate documentation, such as the copy‐paste error, inadequate time to complete notes, interruptions, parallel conversations, or scribe's misunderstanding. The modern approach to clinical record keeping has been the use of the electronic medical record (EMR).^21^ The EMR has demonstrated improved speed of information sharing, increased functionality, and reduced overall hospital costs.^6^, ^22^ Despite these benefits, concerns such as the alteration of team dynamics, shifting attention from the patient, and copy‐paste errors have emerged.^23^ Copy‐paste functionality is useful for the efficiency of documentation and obtaining immediate information pertaining to patients at the patients' bedside.^24^ However, its functionality can result in clinical diagnostic errors and put the patient at risk of harm when false, outdated, or redundant information is propagated from 1 day to the next.^25^, ^26^, ^27^, ^28^ Additionally, this function can add to ‘note bloat’, leading to an unconcise and difficult‐to‐read note that reduces ward round quality.^29^ To adhere to best practice and prevent unnecessary patient harm, it is imperative that patient case notes are complete, accurate, and legible.^18^ The results of this study are in line with previous research demonstrating that ward round documentation should be improved.^21^ Additionally, this study quantifies the extent of documentation inaccuracy and highlights the significant issue of poor surgical ward round documentation.^30^

### Factors affecting accuracy

Patient age was found to significantly affect the accuracy of case notes; the older the patient, the more accurate the case notes were. This may be explained by the clinician perceiving elderly patients as more frail and therefore requiring more care and attention. Although there is little research validating initial clinical impression of frailty, it is often accepted as a useful assessment tool and could result in clinicians taking more care in their documentation.^31^

Secondly, the longer the patient had been admitted to the ward at the time of video recording, the less accurate their case notes were. Patients admitted to the ward for longer periods of time are often complex or have multiple comorbidities which may have impacted the accuracy of documentation. The decline could also be attributable to familiarity between clinician and patient, potentially resulting in an increased chance of human error due to complacency whilst documenting due to cognitive biases. Another possibility is that the copy‐paste functionality of the electronic notes decreased the accuracy, as this function is known to purport inaccurate medical information from 1 day to the next.^25^, ^27^

A main finding of this study was the significant variation between the documentation accuracy of interns scribing. Additionally, the intern documenting the ward round was found to significantly impact the accuracy of documentation in both ‘Major’ and ‘Minor’ points. In the ward round, the scribing intern is tasked to accurately and legibly record all relevant points of discussion into the patient case note in a structured format.^12^, ^13^ This mechanism can fail if the scribe misinterprets information as less important than it is, or if the ward round is time pressured.^1^, ^10^

Interestingly, the experience of the intern (number of days on internship total, number of days on Surgical internship) did not impact the accuracy. This indicates that differences exist between interns in terms of documentation proficiency, likely due to individual differences in factors such as personal confidence, previous related clinical exposure, personal perception of documentation significance, and prior experience with ward round documentation.^32^ The intern has the crucial role of documenting pertinent information in ward rounds.^1^ In Australia, interns often participate in five rotations in different specialties, usually of 10 weeks duration.^33^, ^34^ As a result, they are required to quickly learn and adapt to the new preferences of each unit and the personal preferences of the senior clinicians. While previous literature has demonstrated that interns under‐document important discussions in ward rounds due to the lack of strong evidence‐based training,^30^ results of this study demonstrate that they also incorrectly document or completely omit crucial points of information. To reduce the variation between interns, it is important that senior members of the team prompt engagement and question‐asking behaviours, double‐check and clarify the plan with the scribe, and do so while facilitating environments of psychological safety. There is a general structure and criteria for recording ward rounds, but its application is idiosyncratic, with inclusion criteria varying.^1^ Therefore, to improve the overall documentation, more awareness and documentation‐focused training is needed in surgical education.^35^ This could be achieved with simulated ward rounds and additions to formal medical school curriculums. Artificial intelligence to assist with scribing could also be utilized in the future when the technologies are available.

It is clear that the surgical ward is a complex and chaotic environment and that the job of scribing the ward round is more difficult than leading clinicians might perceive. It is also apparent that consultants may not realize how much of the verbal discussion is not recorded in the notes. Although the senior clinician leading the ward round did not impact the accuracy of case notes, the results of this study indicate that there is an opportunity for them to endeavour to be more supportive of interns to help promote quality documentation. Once a clinician discusses a point, they assume and rely on the intern to document it in the patient's case notes. This assumption, and situational awareness failure to recognize that they have not been heard or understood, is likely causing communication breakdowns, which are known to cause 43% of surgical cases with adverse events.^36^ Clinicians should seek to identify and constantly review the documentation process so that additional guidance, training, and support can be provided. Finally, standardizing elements of the ward round is vital to reduce the variability in documentation accuracy.^15^

The main findings from this study could have significant implications on clinical practice and systems. By using audiovisual recording of the ward rounds and a novel classification system for discussion points (Major, Minor, Not Significant), this study provides insight into the accuracy of ward round documentation which would not otherwise be possible.

## Limitations

There are several potential limitations of this study. The first is the potential of the Hawthorne effect (modifying behaviour in response to being observed)^37^ as staff and patients knew they were being filmed. However, the results of this study demonstrated poor documentation accuracy. If these results were impacted by the Hawthorne effect, it would be expected that the actual accuracy of documentation would be worse when clinicians were not being observed. The use of a convenience sampling technique also may have been a limitation to the study. This sampling technique was used as exclusion criteria eliminated many patients due to being held on precautions. However, this technique is deemed applicable for sampling in clinical research and is appropriate for studies assessing internal validity.^38^ Another limitation of this study may be the different team compositions, whereby different interns were matched with different leading clinicians. Team dynamics can have an impact on understanding, familiarity, and comfort, and needs to be taken into consideration when interpreting these results. However, due to the nature of the surgical ward round, these dynamics could not be controlled. Finally, the scope of this study did not extend into determining whether omitted or incorrect documentation permeated into clinical outcomes.

Despite the limitations, this was the first study investigating the accuracy of ward round documentation by comparing video footage to the patient case note. This study provides an accurate objective measure for measuring this documentation accuracy, and future research should now build on this to determine the impact on patient outcomes.

## Conclusion

Ward rounds are vital to patient care but are incredibly complex. This study has demonstrated that a significant amount of important discussion occurring during surgical ward rounds regarding patient care is not recorded in patient case notes. Inaccurate case notes can be responsible for increased patient complications, longer hospital stays, and greater strain on hospital resources. Future studies should seek to build methodologies ensuring equitable recruitment of patients across multiple specialties. Other patient‐related outcomes such as length of stay, morbidity, and mortality should be investigated against the accuracy of documentation. This study has demonstrated that future interventions to improve the ward round are imperative.

## Author contributions

**Ellie C. Treloar:** Conceptualization; data curation; formal analysis; investigation; methodology; project administration; validation; visualization; writing – original draft; writing – review and editing. **Ying Y. Ting:** Conceptualization; data curation; formal analysis; methodology; visualization; writing – review and editing. **Martin H. Bruening:** Conceptualization; resources; supervision; writing – review and editing. **Jessica L. Reid:** Conceptualization; data curation; methodology; supervision. **Suzanne Edwards:** Formal analysis; resources; software. **Emma L. Bradshaw:** Conceptualization; supervision; writing – review and editing. **Jesse D. Ey:** Writing – review and editing. **Matthias Wichmann:** Conceptualization; supervision; writing – review and editing. **Matheesha Herath:** Conceptualization; writing – review and editing. **Guy J. Maddern:** Conceptualization; funding acquisition; supervision; writing – review and editing.

## Funding information

This study was funded by the Avant Foundation Grant to support initiatives that improve quality, safety, and professionalism in practice. ECT, YYT, MH, and JDE received scholarships from The University of Adelaide and The South Australian Hospital Research Foundation.

## Conflict of interest

Matthias Wichmann and Guy Maddern are Editorial board members of ANZ Journal of Surgery and co‐authors of this article. To minimize bias, they were excluded from all the editorial decision‐making related to the acceptance of this article for publication. The authors have none declared.
